# Effects of Resveratrol‐Loaded Nanoparticles on Follicular Survival, Stromal Integrity and Activity of Endogenous Free‐Radical Scavengers in Bovine Ovarian Cortical Slices Cultured In Vitro

**DOI:** 10.1111/rda.70158

**Published:** 2026-01-28

**Authors:** Mara B. A. Catunda, Francisco das C. Costa, Vitória S. Bezerra, Francisco F. Caetano Filho, Regislane P. Ribeiro, Andreza de A. Silva, Solano D. Martins, Valdevane R. Araújo, Alice V. F. Reis, Josimar O. Eloy, José R. V. Silva

**Affiliations:** ^1^ Laboratory of Biotechnology and Physiology of Reproduction Federal University of Ceará Sobral Ceará Brazil; ^2^ Reproductive Physiology Research Laboratory Federal University of Delta do Parnaíba ‐ UFDPar Parnaíba Piauí Brazil; ^3^ Center for Pharmaceutical Development and Testing Federal University of Ceará Fortaleza Ceará Brazil

**Keywords:** bovine, follicles, nanoparticles, resveratrol

## Abstract

The aims of this study were to assess the effects of resveratrol‐loaded nanoparticles (RLNP) on follicular survival, stromal integrity and activity of endogenous free‐radical scavengers in bovine ovarian tissues cultured in vitro. Ovarian cortical slices were incubated in α‐MEM^+^ alone or supplemented with 0.2, 2.0 or 20.0 μM RLNP, blank nanoparticles (BNP) or free resveratrol (20.0 μM) for 6 days at 38.5°C and 5% CO_2_. Follicular integrity, number of stromal cells, density of collagen fibres, levels of thiol and activity of free‐radical scavengers (glutathione peroxidase [GPX], superoxide dismutase [SOD] and catalase [CAT]) were evaluated in tissues cultured in the different treatments. The data showed that ovarian cortex cultured with 20.0 μM free resveratrol or RLNP, in all tested concentrations, had a reduced rate of morphologically intact follicles in relation to uncultured controls (*p* < 0.05). The RLNP (0.2, 2.0 or 20.0 μM) and BNP increased the proportion of growing follicles and stromal cell numbers (*p* < 0.05). Collagen fibre levels decreased in tissues cultured with 0.2 or 2.0 μM RLNP compared to uncultured controls, but remained greater than those seen in ovarian cortex cultured in other treatments (*p* < 0.05). Free resveratrol increased CAT and GPX activity, while RLNP reduced activity of SOD and GPX (*p* < 0.05). In conclusion, RLNP improved follicle survival and growth, preserved stromal tissue and modulated the activity of free‐radical scavengers in bovine ovarian slices cultured in vitro.

## 1 Introduction

Several researchers have focused on developing in vitro culture systems that support the survival and growth of early‐stage follicles enclosed within ovarian tissue (Telfer and Andersen 2021). Since primordial follicles represent the largest oocyte reservoir, their in vitro growth offers a valuable opportunity to increase the number of fertilizable oocytes for use in animal reproductive technologies (Taghizabet et al. 2022). However, the efficiency of culture systems of early ovarian follicles can be negatively affected by in vitro excessive levels of reactive oxygen species (ROS) (Silva et al. 2023). This exposure can lead to mitochondrial damage, lipid peroxidation and compromised cell integrity, ultimately resulting in reduced follicle growth, survival and oocyte integrity (Paulino et al. 2022; Song et al. 2024). Therefore, controlling oxidative stress is a key challenge for maintaining follicular health and tissue homeostasis in vitro.

Natural antioxidants have been proposed as potential agents to mitigate redox imbalance during in vitro culture of ovarian tissues, and plant‐derived compounds have shown strong potential to reduce free radical‐induced damage in vitro (Gaviria et al. 2019). Resveratrol (3,5,4′‐trihydroxystilbene) is a polyphenol found in various plants that has received particular attention due to its antioxidant, and antiapoptotic effects (Meenakshi et al. 2024; Ortega et al. 2012). Recently, Costa et al. (2025) showed that resveratrol helped to preserve ovarian stroma and to keep follicular health in cultured ovarian slices. However, the direct use of resveratrol in biological systems is limited by its photo‐instability, pH sensitivity and poor solubility, which collectively can reduce its bioavailability and antioxidant efficiency (Liu et al. 2018).

Nanotechnology‐based delivery systems have emerged as a promising strategy to overcome these limitations. Polymeric nanoparticles, in particular, can improve drug stability and solubility, prolong circulation time, and enable controlled release of bioactive compounds (Annaji et al. 2021; Freitas et al. 2023). By encapsulating resveratrol, these carriers can enhance its interaction with cells, protect against premature degradation, reduce the required dosage, and optimise its antioxidant performance (Pontes‐Quero et al. 2021). Incorporating such nanocarriers into the formulation of culture media could lead to optimised systems capable of sustaining redox homeostasis and preserving the structural integrity of ovarian tissues during in vitro culture. It is hypothesized that nanoencapsulated resveratrol can modulate redox balance and extracellular matrix remodelling in ovarian cortical tissue and, consequently, positively affect follicular activation, survival and stromal integrity.

This work aimed to investigate the influence of resveratrol‐loaded nanoparticles (RLNP), at different concentrations, on follicular survival, stromal integrity and activity of endogenous free‐radical scavengers (GPX, SOD and CAT) in bovine ovarian tissues cultured in vitro.

## 2 Materials and Methods

### 2.1 Chemical Products and Preparation of Resveratrol‐Loaded Nanoparticles

Resveratrol was supplied by Suzhou Vitajoy (Suzhou, China). The reagents and cultured medium used in this research were acquired from Sigma‐Aldrich (St. Louis, MO, USA), except where otherwise stated in the text. The preparation of resveratrol‐loaded (RLNP) or blank nanoparticles (BNP) was performed according to the protocol described by Freitas et al. (2023). The analysis focused on three factors: size, polydispersity index (PDI) and zeta potential. The distribution of sizes in terms of hydrodynamics, zeta potential and PDI of the nanoparticles were investigated at 25°C.

### 2.2 Source of Ovaries and Ethical Approval

Ten pairs of ovaries from five adult, cyclic cows were obtained from a local abattoir. After collection, the ovaries were first rinsed in 70% alcohol for 10 s, followed by two washes in sterile 0.9% saline solution with antibiotics. The ovaries were transported to the laboratory at 4°C in 0.9% saline solution with antibiotics within 1 h. This research was approved by the Ethics and Animal Welfare Committee (Federal University of Ceará, number 04/2022).

### 2.3 In Vitro Cultivation of Bovine Ovarian Cortical Slices

Under sterile conditions, 3 × 3 × 1 mm ovarian cortical fragments were recovered from the pair of ovaries and immediately transferred to a dissection medium, that is, α‐MEM supplemented with 100 μg/mL streptomycin and 100 μg/mL penicillin at 38.5°C. For the uncultured control group, four cortical fragments from each ovarian pair were fixed in 4% paraformaldehyde for 24 h at room temperature prior to histological analysis. The remaining ovarian cortical slices were cultivated in vitro in 24‐well plates. The α‐MEM was the culture medium, to which 2 mM of hypoxanthine, 2 mM glutamine, 5.5 μg/mL transferrin, 10 μg/mL insulin, 5 ng/mL selenium, 1.25 mg/mL bovine serum albumin, 100 μg/mL streptomycin and 100 μg/mL penicillin were added (α‐MEM^+^). The cortical slices were cultured in 500 μL of α‐MEM^+^ alone or supplemented with 0.2, 2.0 or 20.0 μM RLNP, as well as with 20.0 μM BNP or 20.0 μM resveratrol not associated with nanoparticles (RSV). These concentrations were selected according to Rocha et al. (2018). Every 2 days, 60% of culture medium was refreshed. We repeated each treatment five times. After the culture period ended, the fragments were processed for histological analysis to assess follicular morphology and growth, collagen in the ECM and the number of stromal cells. The activity of antioxidant enzymes was also assessed.

### 2.4 Histological Analysis

Uncultured or cultured ovarian slices were fixed in 4% paraformaldehyde and processed for classical histology. Then, 6.0 μm sections were stained with haematoxylin and eosin. The slides were analysed using a Nikon microscope at 100× and 400× magnification. Follicles were classified as primordial, primary and secondary follicles as previously described by Figueiredo and Lima (2017). Follicles with a healthy oocyte surrounded by well‐organised granulosa cells and no signs of nuclear pyknosis were classified as morphologically intact. In contrast, follicles exhibiting oocyte shrinkage, disorganised granulosa cells and/or pyknotic nucleus were considered degenerated. The rate of healthy follicles, as well as at different stages of development, were calculated in relation to uncultured control and after 6 days of in vitro cultivation.

### 2.5 Analysis of Ovarian Stromal Cells and Collagen Content

The analysis of stromal integrity was assessed in both uncultured and cultured ovarian cortical tissues. Stromal cells were quantified in a 100‐μm^2^ area within 10 fields from different sections of histological slides from five animals. The quantification of stromal cells per field was performed using the method reported by Cavalcante et al. (2019). A single operator carried out all of the analysis. To evaluate collagen fibres, the tissue sections were stained with Picrosirius Red (Abcam Kit) according to Rittié (2017). In brief, 6‐μm‐thick ovarian sections were incubated in a 0.1% Sirius Red solution for 1 h. Then, the sections were dehydrated and mounted on slides. They were subsequently observed under a Nikon Eclipse TS 100 optical microscope at 400× magnification. Ten images of collagen fibres from different sections of ovarian tissue**s** and the density of collagen fibres were evaluated by using ImageJ software (version 1.51p, 2017).

### 2.6 Total Proteins, Thiol Levels and CAT, SOD and GPX Activity

For these analyses, samples of 100 mg/mL from tissues cultured under different treatment conditions were macerated in a potassium phosphate buffer. The homogenates were centrifuged at 1500 *g* for 10 min at 4°C, and the resulting supernatant was collected as previously described by Ellman (1959). Quartz cuvettes (Thermo Fisher Scientific) were used for these assays. The data are expressed as the mean ± SEM of enzyme units per milligram of protein. The Bradford method was used to determine protein concentration (Bradford 1976). This method uses Coomassie Blue (Quick Start/Bradford; Bio‐Rad) to measure total protein concentration in ovarian tissue samples. Total protein concentration was assessed using spectrophotometry at a wavelength of 595 nm and a standard curve with albumin concentrations ranging from 0 to 50 mg/mL. The albumin curve was then used to standardise pro (thiol) and antioxidant (SOD, CAT and GPX) content levels.

The total thiol content of the ovarian slices was quantified by using 5,5′‐dithiobis (2‐nitrobenzoic acid) (DTNB; Sigma) as a reduced thiol index. At a neutral pH, thiol residues react with 10 mM DTNB, cleaving the disulfide bond to form the 2‐nitro‐5‐thiobenzoate anion. The DTNB is then quantified using a spectrophotometer at a wavelength of 412 nm (Takahashi et al. 1978).

The measurement of CAT, SOD and GPX activity was performed as described by Costa et al. (2025). Briefly, the activity of SOD was quantified by inhibiting the auto‐oxidation of adrenaline at 480 nm every 10 s for 180 s. The activity of CAT was quantified through the consumption of hydrogen peroxide (H_2_O_2_) as a substrate at 240 nm. Absorbance was recorded twice every 30 s. The GPX activity was assessed by monitoring the NADPH oxidation, quantified as the reduction in absorbance at 340 nm, being evaluated every 10 s for 300 s.

### 2.7 Statistical Analysis

The percentage of intact follicles, as well as of primordial and developing follicles in each treatment was evaluated using the chi‐squared test (GraphPad Prism software, version 9.0). The distribution of collagen fibres and stromal cells, and the activity of free‐radical scavengers were analysed using ANOVA and the Kruskal–Wallis test (*p* < 0.05).

## 3 Results

### 3.1 Physicochemical Characterisation of Resveratrol‐Loaded Nanoparticles

The size of nanoparticles was characterised by dynamic light scattering and the data showed RLNP and BNP had 135.93 and 134.3 nm, respectively (Table 1). Data on PDI and zeta potential of RLNP and BNP are shown in Table 1.

**TABLE 1:** Physicochemical characterisation of the formulations in relation to particle size, polydispersity index (PDI) and zeta potential.

Formulation	Size (nm)	PDI	Zeta potential (mV)
BNP 1	133.3	0.187	−2.01
BNP 2	134.2	0.202	−2.65
BNP 3	135.4	0.201	−4.17
Mean ± SD	134.3 ± 1.05	0.197 ± 0.01	−2.94 ± 1.11
RLNP 1	132.6	0.161	−2.03
RLNP 2	137.7	0.182	−2.47
RLNP 3	137.5	0.217	−4.2
Mean ± SD	135.93 ± 2.89	0.187 ± 0.03	−2.90 ± 1.15

Abbreviations: BNP, blank nanoparticles; RLNP, resveratrol‐loaded nanoparticles.

### 3.2 Effects of RLNP on Follicular Survival and Growth

**FIGURE 1:**
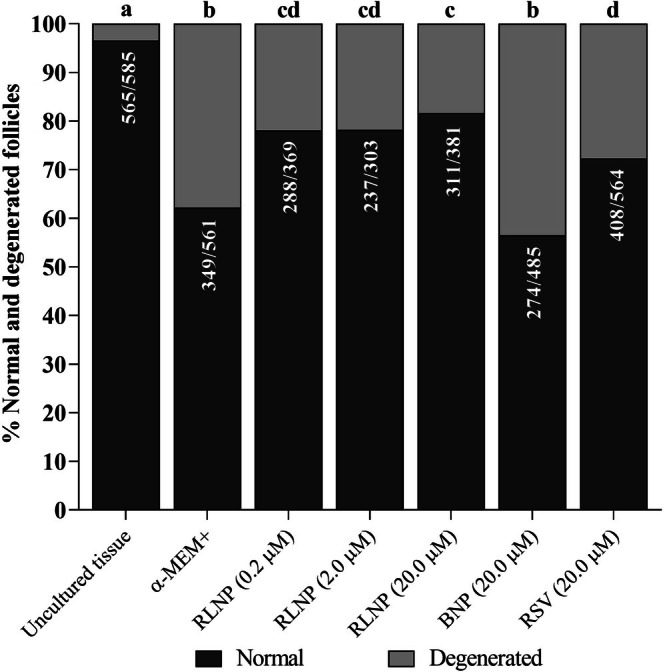
Rate of intact and degenerated follicles before and after culturing ovarian tissues in a control medium (α‐MEM^+^) with or without the addition of 0.2, 2.0 or 20.0 μM of RLNP, BNP or free resveratrol. Letters (a–d) show differences among treatments (*p* < 0.05).

**FIGURE 2:**
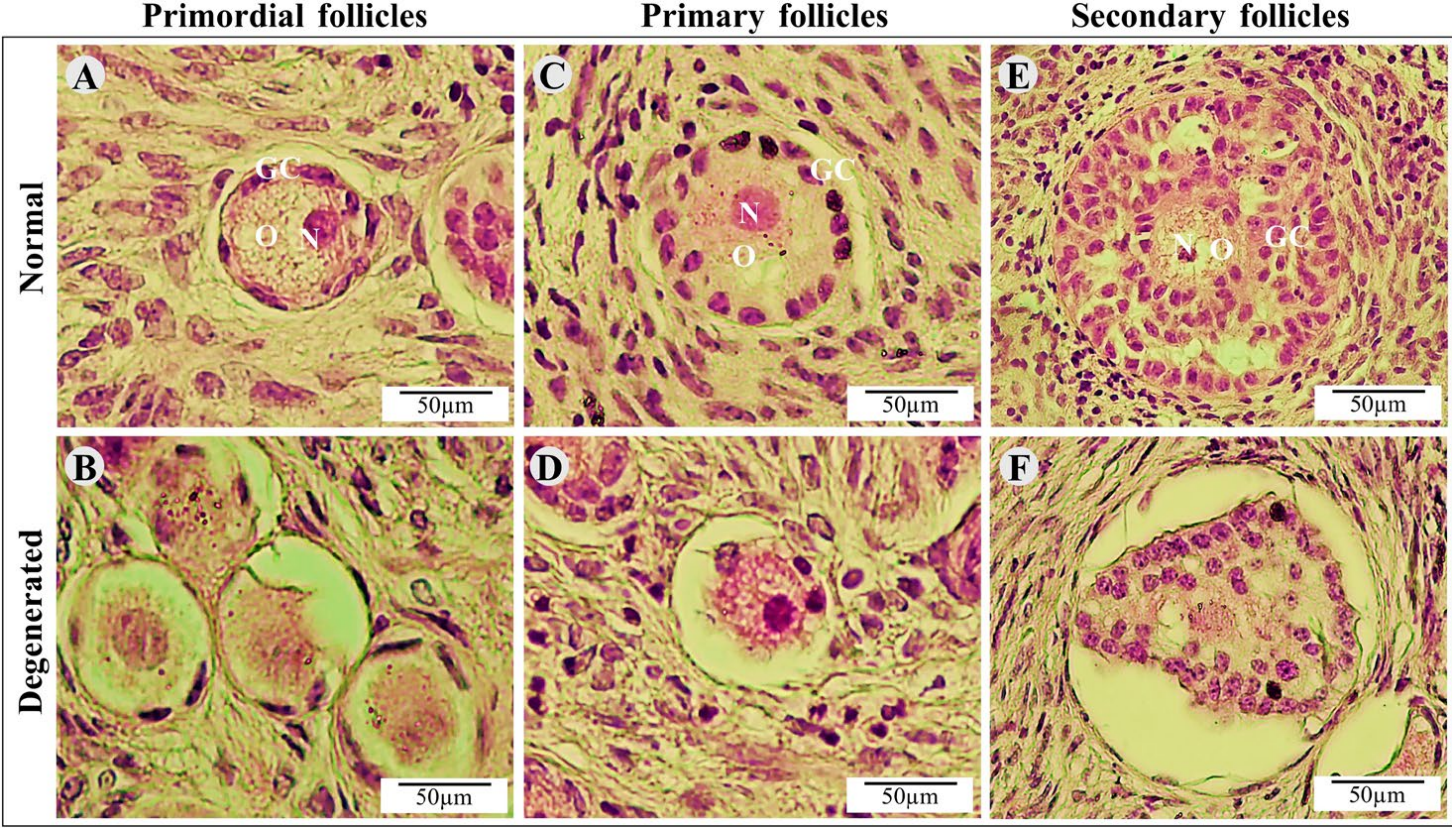
Morphology of normal (A, C, E) and degenerated (B, D, F) primordial, primary, secondary follicles. GC, granulosa cells; N, nucleus; O, oocyte. Scale bar: 50 μm.

**FIGURE 3:**
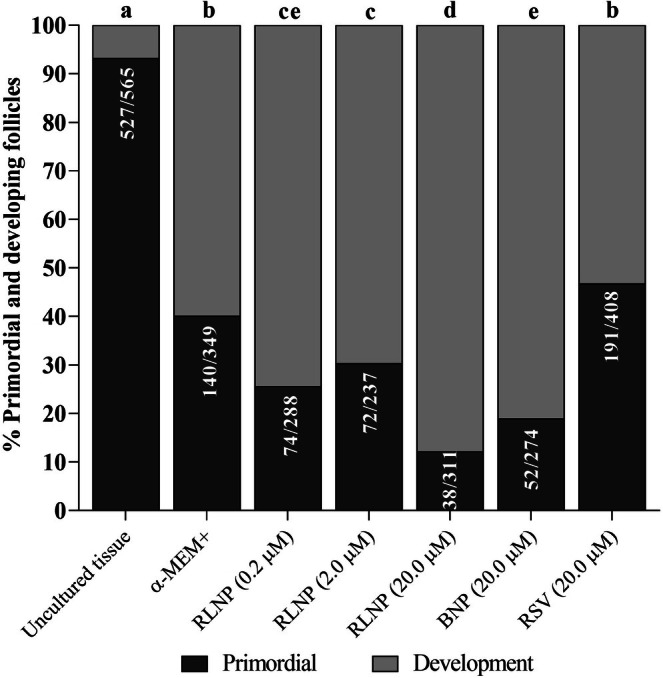
The rate of primordial and developing follicles in uncultivated ovarian slices and in slices incubated in control medium (α‐MEM^+^) alone or supplemented with 0.2, 2.0 or 20.0 μM BNP and/or free resveratrol for 6 days. Letters (a–d) indicate differences among treatments (*p* < 0.05).

At day 6, tissues cultivated in all treatments exhibited a lower rate of intact follicles in comparison with uncultured tissues. Nonetheless, the presence of RLNP in culture medium, at all concentrations tested, increased the percentages of intact follicles when compared to tissues cultured with BNP or free resveratrol (Figures 1 and 2). Regardless of the treatment, cultured ovarian tissues exhibited a higher proportion of growing follicles than uncultured tissues. Additionally, tissues cultured with 20.0 μM RLNP had a lower percentage of primordial follicles and significantly greater rate of development follicles in relation to ovarian cortex cultured in other treatments (*p* < 0.05). In addition, tissues cultured with free resveratrol were not different from those incubated in control medium (Figure 3).

### 3.3 Effects of RLNP on Ovarian Cortex Stromal Integrity

**FIGURE 4:**
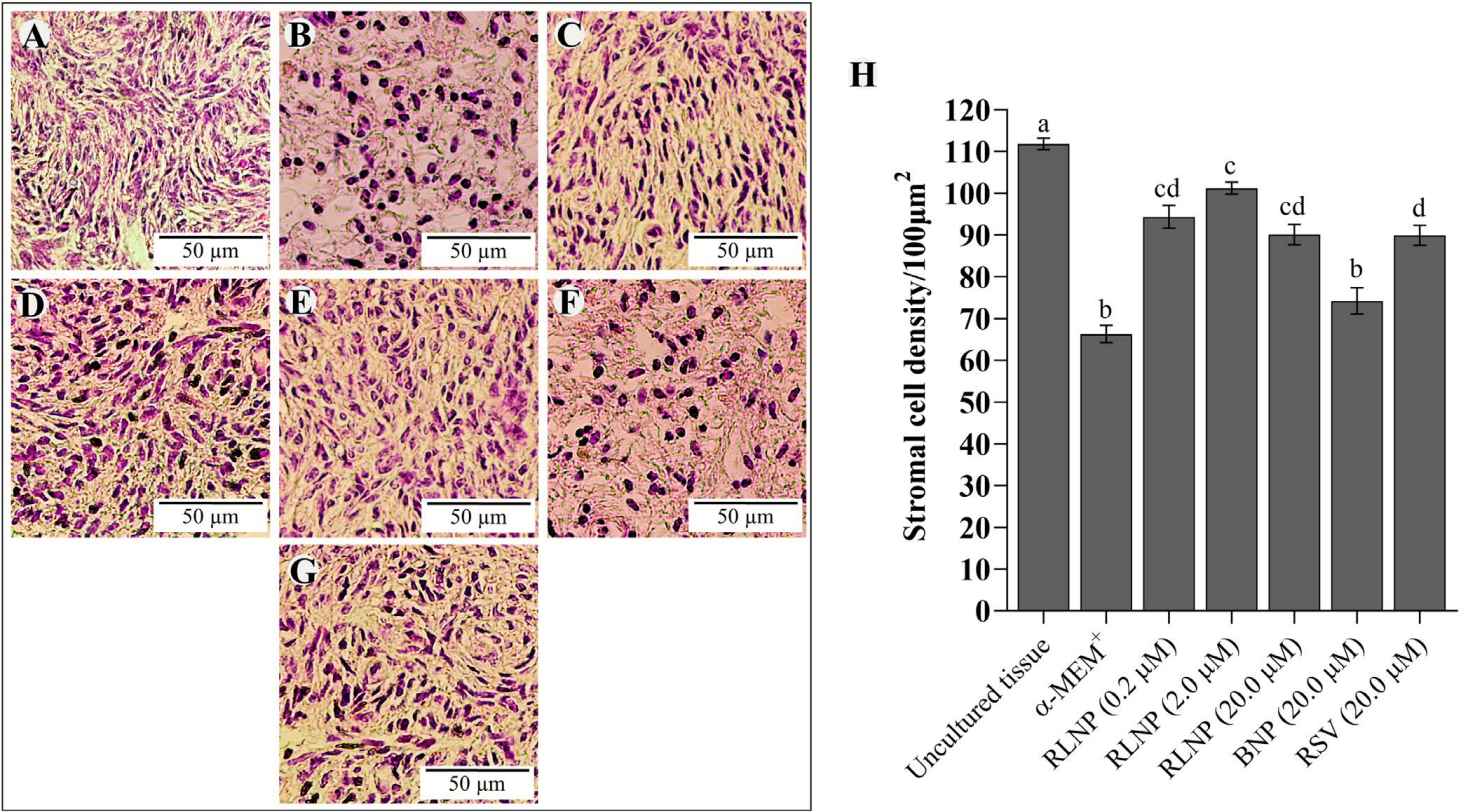
Number of stromal cells uncultivated ovarian slices (A) and in those cultivated in MEM^+^ alone (B) or supplemented with 0.2 (C); 2.0 (D); 20.0 μM RLNP (E); 20.0 μM BNP (F) or 20.0 μM RSV (G). The number of cells in slices cultivated in each treatment is shown in H. Scale bar: 50 μm. Letters (a–d) indicate differences among treatments (*p* < 0.05).

**FIGURE 5:**
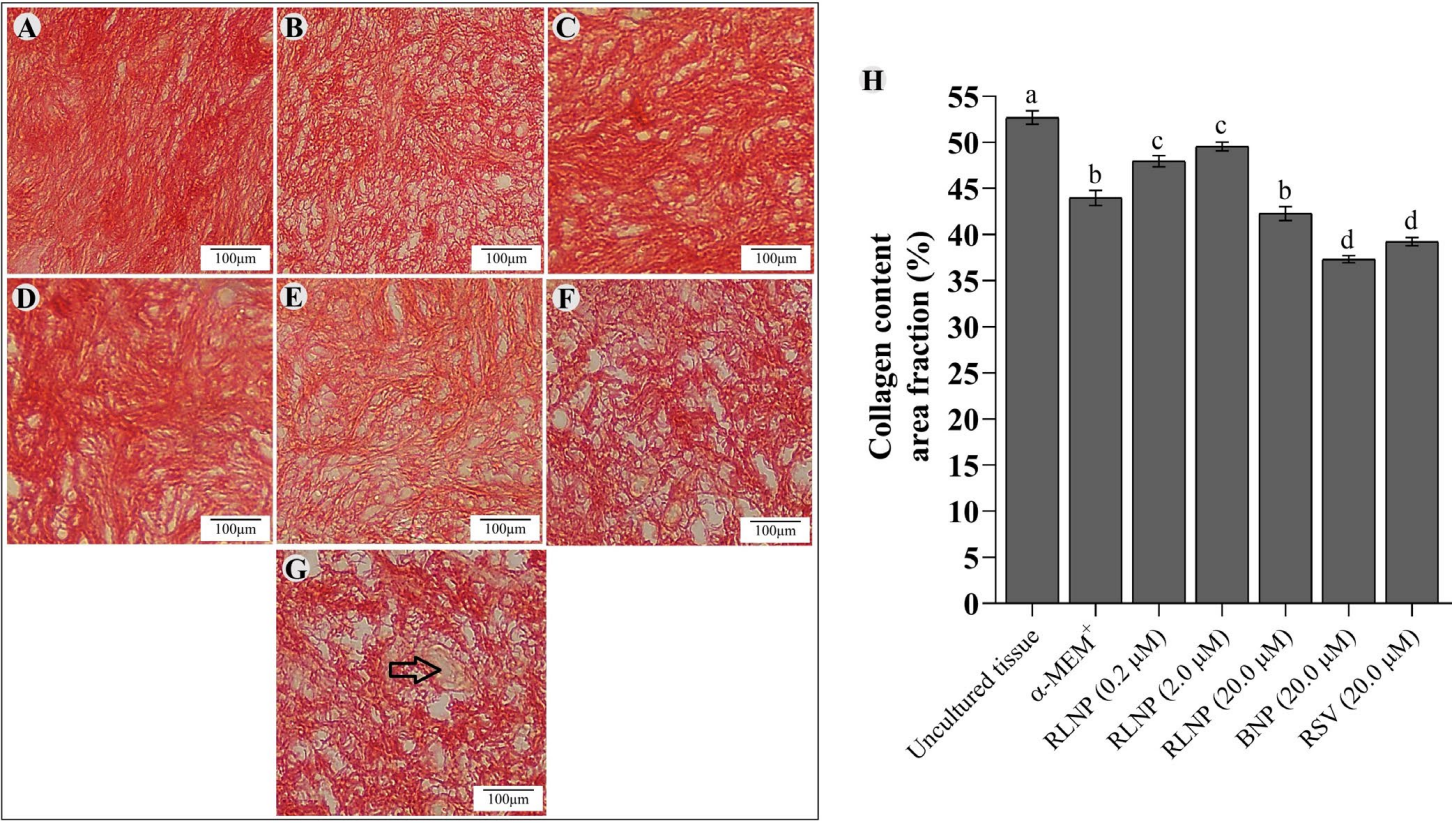
The collagen fibres are shown in uncultivated ovarian slices (A), and in slices cultivated in α‐MEM^+^ alone (B) or supplemented with 0.2 μM RLNP (C), 2.0 μM RLNP (D), 20.0 μM RLNP (E), 20.0 μM BNP (F) or 20.0 μM RSV (G). The black arrow shows an uncoloured follicular area (G). The rate of collagen in uncultivated and cultivated ovarian slices is shown in H. Scale bar: 100 μm. Letters (a–d) indicate differences among treatments (*p* < 0.05). Arrow indicates unstained ovarian follicle.

Ovarian cortex cultured in all treatment had a significantly lower number of stromal cells than uncultured tissues (*p* < 0.05). Tissues cultured in medium supplemented with RLNP had, however, a significantly higher number of stromal cells than those cultivated in α‐MEM^+^ alone or in presence of BNP. The stromal cell survival was greater in tissues cultured with 2.0 μM RLNP than that seen in tissues cultured with free resveratrol (*p* < 0.05) (Figure 4). At day 6, the collagen fibre content of tissues cultured in all treatments had decreased compared to uncultured tissues. However, cortical slices cultured with 0.2 or 2.0 μM RLNP exhibited higher collagen fibre levels than those cultivated in control medium (α‐MEM^+^) or in this same medium supplemented with 20.0 μM RSV, BNP or free RSV (Figure 5).

### 3.4 Levels of Thiol and Activity of Free‐Radical Scavengers

**FIGURE 6:**
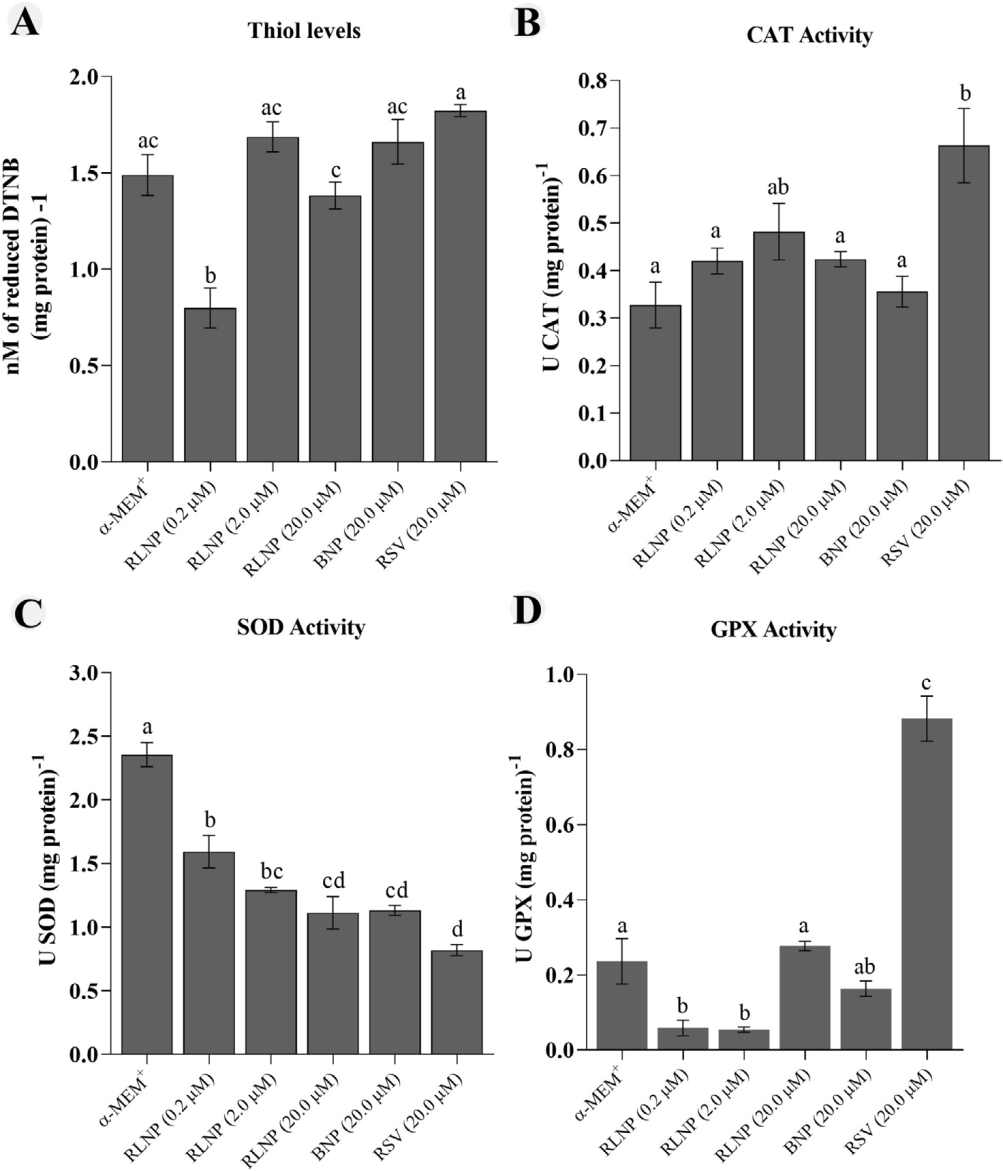
Thiol levels (A) and CAT (B), SOD (C) and GPX (D) activity in ovarian slices cultivated in control medium (α‐MEM^+^) alone or supplemented with 0.2, 2.0 or 20.0 μM RLNP or 20.0 μM BNP and 20 μM free resveratrol. Different lowercase letters (a–d) indicate differences between treatments (*p* < 0.05).

Tissues cultured with 0.2 μM RLNP exhibited significantly lower thiol levels than those cultivated in other treatments (*p* < 0.05) (Figure 6A). Ovarian slices cultured with free resveratrol demonstrated significantly higher CAT activity than those cultured in other treatments, except those cultured with 2.0 μM RLNP (Figure 6B). Regardless of the treatment, all concentrations of RLNP, BNP or free resveratrol reduced SOD activity after 6 days of culture in comparison to ovarian cortex cultivated in control medium (Figure 6C). The GPX activity was lower in slices cultivated with 0.2 or 2.0 μM RLNP than those cultivated in control medium. In contrast, 20.0 μM free RSV significantly increased GPX activity (*p* < 0.05) (Figure 6D).

## 4 Discussion

This is the first study to show that RLNP increases the proportion of morphologically intact follicles in in vitro cultured ovarian cortical tissues. Notably, RLNP was more effective to protect ovarian follicles than free resveratrol. In a previous study, resveratrol encapsulated in nanoparticles had a spherical shape and mean diameter of 145 nm, indicating that the synthesis method is robust and capable of producing nanoparticles (Freitas et al. 2023). Our findings are in line with Li et al. (2023), who demonstrated that nanoparticle incorporation enhances resveratrol bioavailability and stability. Nanoparticles enable sustained and targeted delivery (Lee et al. 2022), ensuring continuous cellular protection. Previous studies showed that resveratrol protects cells against DNA fragmentation and promotes proliferation (ovine; Bezerra et al. 2018), and prevents apoptosis (murine; Ortega et al. 2012) in cultured granulosa cells, as well as downregulates pro‐apoptotic genes in oocytes and cumulus cells (porcine; Kwak et al. 2012). Moreover, resveratrol has been shown to improve follicle survival by regulating anti‐inflammatory and antioxidant pathways (Wang et al. 2021).

Supplementing the culture medium with 20.0 μM of RLNP, but not with free resveratrol, increased the proportion of activated follicles in relation to tissues cultivated in control medium. Consistent with our findings, a higher rate of developing follicles was also seen in human ovarian slices cultivated with resveratrol (Hao et al. 2018). Resveratrol stimulated granulosa cell proliferation (Bezerra et al. 2018), increased PCNA levels in porcine cumulus cells during in vitro maturation (Kwak et al. 2012), and enhanced thymidine incorporation in murine granulosa cells, indicating mitogenic activity (Ortega et al. 2012). Additionally, resveratrol may influence granulosa cell metabolism, supporting oocyte development through the energetic metabolism crosstalk between oocyte and granulosa cells (Ragonese et al. 2021).

This study revealed a marked reduction in the collagen fibre area across all cultured tissues compared to uncultured control, which is in line with previous reports showing ECM degradation during in vitro culture (Quan et al. 2013). Matrix metalloproteinases‐2 and ‐9 are central to ECM degradation, and their expression is upregulated under oxidative stress (Verma and Hansch 2007; Ali et al. 2024). It is important to note that tissues cultivated with 0.2 and 2.0 μM RLNP had higher levels of collagen fibres and consequently greater tissue stiffness than those seen in tissues cultured in other treatments. Ovarian rigidity plays a substantial role in the maintenance of follicular quiescence and growth (Shah et al. 2018). Primordial follicles are located in the stiffest region of the outermost cortex, whereas growing follicles are more commonly found in less rigid regions (Hornick et al. 2012). The survival of follicles and their appropriate growth depend on the ovarian rigidity gradient (Hornick et al. 2012). Collagen fibres provide structural support and exert a crucial role in cell signalling and tissue homeostasis (Verma and Hansch 2007; Amargant et al. 2020).

The RLNP increased stromal cell numbers during in vitro cultivation of bovine ovarian cortical slices in comparison to slices cultivated in control medium. This may be due to the greater bioavailability of encapsulated resveratrol and its modulatory effects on ovarian cell survival (Long et al. 2020; Naserifar et al. 2020). Previous research also reported a beneficial influence of resveratrol on acinar cell density (Alves et al. 2017) and bone density (Wong et al. 2020). Stromal cells are known to support cell growth by creating a permissive microenvironment that regulates development, steroidogenesis, morphology and intercellular communication (Grosbois et al. 2023). In this context, we suggest that RLNP can positively influence stromal density, potentially enhancing follicular development.

Tissues cultured with RLNP had reduced SOD activity, which reflects its ability to reduce superoxide levels, thereby decreasing the need for SOD activity (Liang et al. 2023). These data are consistent with previous studies that emphasised the ability of resveratrol to reduce ROS levels during the in vitro maturation of oocytes in bovine species (Wang et al. 2014). The increase of CAT and GPX in tissues cultured with free resveratrol aligns with studies reporting that free resveratrol is a potent inducer of free‐radical scavengers, such as CAT (Ren et al. 2019), possibly via activation of SIRT1 (Franco et al. 2023). The increased GPX activity in tissues cultured with free resveratrol can be related to activation of the Nrf2 pathway, as described previously in leukocytes by Franco et al. (2023). In contrast, RLNP reduced GPX activity compared to free resveratrol, possibly due to lower ROS levels, as also observed for SOD. Additionally, 0.2 μM RLNP reduced tissue thiol levels, indicating decreased availability of reducing compounds, which are markers of oxidative stress and cellular protection. These results show that the oxidative protection of RLNP involves redox modulation (Morita et al. 2012). Notably, free resveratrol maintained higher thiol levels than those seen in tissues cultured with 0.2 μM RLNP, supporting the hypothesis that encapsulation enhances bioavailability and therapeutic efficacy (Freitas et al. 2023).

## 5 Conclusions

The supplementation of culture medium with 2.0 and 20.0 μM RLNP has a positive impact on maintenance of follicular health and ovarian stromal integrity. The RLNP reduces antioxidant enzyme activity and thiol levels and modulates oxidative stress in cultured ovarian cortical slices in bovine species. This study proposes a new culture system that integrates nanotechnology and in vitro ovarian follicle development, providing fundamental evidences to support future advances in assisted reproductive technologies.

## Author Contributions

**Mara B. A. Catunda:** writing – original draft, methodology, data curation. **Francisco das C. Costa:** methodology, data curation, formal analysis. **Vitória S. Bezerra:** methodology, writing – review and editing. **Francisco F. Caetano Filho:** methodology. **Regislane P. Ribeiro:** methodology. **Andreza de A. Silva:** methodology, writing – review and editing. **Solano D. Martins:** methodology. **Valdevane R. Araújo:** methodology. **Alice V. F. Reis:** methodology. **Josimar O. Eloy:** supervision, investigation, writing – review and editing. **José R. V. Silva:** supervision, investigation, writing – review and editing, project administration, funding acquisition, conceptualization.

## Funding

This study was supported by the National Council for Scientific and Technological Development (CNPq, Brazil, Grant number 403862/2024‐8).

## Conflicts of Interest

The authors declare no conflicts of interest.

## Data Availability

The data that support the findings of this study are openly available in Universidade Federal do Ceara at https://repositorio.ufc.br/.
